# More landings for higher profit? Inverse demand analysis of the bluefin tuna auction price in Japan and economic incentives in global bluefin tuna fisheries management

**DOI:** 10.1371/journal.pone.0221147

**Published:** 2019-08-23

**Authors:** Chin-Hwa Sun, Fu-Sung Chiang, Dale Squires, Anthony Rogers, Man-Ser Jan

**Affiliations:** 1 Institute of Applied Economics, National Taiwan Ocean University, Keelung, Taiwan, Republic of China; 2 Department of Economics, University of California San Diego, La Jolla, California, United States of America; 3 California Ocean Science Trust, Oakland, California, United States of America; Facultad de Ciencias Empresariales y Turismo, SPAIN

## Abstract

This paper estimates the price changes in global bluefin tuna (BFT) markets in response to shifts in regional and global landings to evaluate the conservation and economic incentives from changes in the Total Allowable Catch (TAC) managed by all three Regional Fisheries Management Organizations. A fisherman’s income, and thus the financial incentive to accept management measures controlling catch levels, depends in part on how responsive price is to overall catch. Individual fisherman, with their own best interest in mind, used to wish to increase their individual landings and create an incentive to ask to increase the TAC for the industry, without realizing the possible revenue loss due to the resulting falling prices. To protect the value of all stakeholders’ property rights, a consensus to avoid abruptly raising the TAC, without first considering the potential loss due to market response, is needed. Alternatively, if revenue increases with lower TAC, a positive economic incentive for conservation is created if price increasing proportionately more than the lower supply, with harvest profits boosted by lower costs of production. To capture the complexity of substituting across various sources of supply and product form, a general synthetic inverse demand system is estimated to identify the impact of overall landings on BFT prices. This system estimates price flexibilities of both fresh and frozen longline-caught sashimi-grade tunas (Pacific, Atlantic and southern bluefins, and bigeye) at the Tokyo Center Market in Japan, including the Tsukiji Market, the world’s largest fish auction market that served as the single global price leader for BFT. The resulting estimation shows that own-quantity price flexibilities of every type of fresh and frozen BFTs are less than unity and inflexible in their own consumption. This creates poor individual producer incentives for fishermen to reduce wild or farmed BFT supply, as there is a chance to increase their own revenue, under the unlikely condition that the total supply is fixed. However, by observing the rapid increases in the TAC of Eastern Atlantic bluefin tuna (EABFT) in the coming years, suppliers may not be better off as price will drop proportionally faster and total revenue if the estimated scale flexibility is greater than one. Based on the estimated scale flexibility of frozen BFT, which is slightly less than unity, the frozen subsector of EABFT suppliers is the only winner under the supply increases. Suppliers of frozen BFT in other regions, fresh BFT (in the Atlantic and elsewhere), and southern BFT and bigeye tuna will all be harmed through lower revenue by the supply increases. Additionally, while total revenue might stay the same for frozen BFT suppliers, fishermen will potentially receive lower profits due to higher operating costs associated with increased landings when the supply of EABFT increases. Given the number of sectors that ultimately lose financially in the short term and given the ecological (and production) risks accompanying an abrupt increase in fishing pressure in the long term, the global economic losses resulting from an increase in the allowable catch of Atlantic bluefin tuna will outweigh any potential increases to revenue.

## Introduction

One of the primary challenges in fisheries management is to determine the right set of incentives for resource conservation and management of the industry, which depends on the utilization of its resource. Fisheries managed by a Total Allowable Catch (TAC) and not covered by individual or group property rights are driven by the “race to fish.” The tragedy of the commons externality creates incentives among harvesters to expand the TAC and allow more catch, ceding higher revenues and profits to harvesters. However, is expanding the TAC always a preferred solution? Does an increase in TAC truly guarantee an increase in revenues and profits? After all, raising the TAC can also lower the resource stock and thus the long-run, sustainable supply.

Furthermore, are there other economic incentives that arise from TAC-managed fisheries under open access in addition to the “race to fish”? Are there related management options? The answers depend, in part, on the nature of the product’s price response to changes in TAC. TAC, in this case, is the aggregate supply in the market in which product prices form. Depending on the responsiveness of prices to declines in quantities, reductions in TAC that favor conservation might increase prices more than they cause a fall in quantity. In this case, the accompanying revenue increase would compensate for the decrease in quantity under a reduced TAC. Access fees of coastal states can also potentially be raised [1]. A lower TAC and larger resource stock following population growth can also increase society’s welfare as measured by non-market economic values, such as increased biodiversity and ecosystem services (indirect use values) and greater assurance of the continued existence of a species and richer ecosystem (existence value). In short, conservation, in circumstances when reduced overall catch increases prices, can increase economic rents and generate positive economic incentives for conservation that enhance acceptance of management measures and cooperation, i.e., conservation can be profitable.

Besides economic rent (producer surplus, economic profits), the other half of total economic benefits is consumer benefits (consumer surplus or the more preferred measures of compensating or equivalent variation). Part of the increase in producer benefits with a higher price and lower quantity comes from a transfer from consumer benefits. To the extent that the inverse demand curve estimated in this paper is an equilibrium demand curve, the welfare measures capture both consumer and producer surplus [2]. Even when consumer benefits from direct use values decline due to a rise in price and fall in quantity, consumers can gain through increased enjoyment of non-market values such as indirect use value and existence value when there is more conservation from larger resource stocks. In short, the consumer picture is more complex and consumer gains in non-market benefits can potentially outweigh any reduction in consumer benefits from the bluefin market’s higher prices. Conversely, increases in TAC can almost paradoxically lower producer revenues and profits.

As early as 1696, Gregory King [3] had shown how an abundant harvest would dramatically reduce a farmer’s revenue, long before Augustin Cournot [4] and Alfred Marshall [5] specified the conditions under which King’s law holds, i.e., prices falling proportionately more than the proportionate increase in supply. However, the fishing industry is generally less concerned about a scenario like King’s foundational example—a price flexibility of demand greater than unity in absolute value. Given the persistent challenges posed by overfishing and excess effort, fisheries managers are typically far more concerned with the consequences of catch reduction than expansion, at the current level of fish stocks [1]; [6–9].

In addition to the efficacy of an expanded TAC, two related questions can be asked: are the gains from rights-based management due to the incentives created by secure and transferable rights, or due to a more conservative or better-enforced TAC? And are there additional unrecognized–and even unused–economic incentives available to fisheries managers, aside from the TACs (regardless of whether or not there are some types of property rights)? (This issue extends to many cap-and-trade systems.) These questions have only rarely been raised or thoroughly investigated in the fisheries and property rights literature [10] [11]. While a better understanding of the price responsiveness to changes in both quantities landed and TACs cannot directly answer the second question, we can unequivocally state that price responsiveness leading to declines (increases) in revenues with reduced quantities creates incentives that counter (reinforce) the gains in positive incentives from rights-based management [12]. A related issue is the proportionate change in costs. With constant returns of scale proportionate changes in profits or economic rent track changes in revenues. Unitary price flexibilities lead to no changes in revenues whether the TACs are expanded or contracted.

In sum, economic incentives created through product price responsiveness have substantial effects on conservation, industry profits, and society’s economic rent, but remain an overlooked policy consideration. When price rises more than a proportional decrease in supply, thereby affecting revenue and profit more than proportionately, an additional conservation incentive is created: conservation pays. When price falls proportionately more than the increase in TAC, industry has an economic incentive to act collaboratively and discourage the increase in TAC to protect the profitability of their fishery

Evaluating these economic incentives under a TAC system (or any industry quota/cap on overall output) requires information on the product price responsiveness, calling for the estimation of a demand system. To that end, a series of choices must be made before a good model specification can be reached. The demand function can either be linear or logarithmic, ordinary or inverse, final or derived, Marshallian or Hicksian, static or dynamic, detailed or aggregated, etc. These options are not neutral and can lead to substantial differences in the empirical estimates of how the prices of a product responds to a change in its own quantity (“price flexibility”) and to a change in all of the combined quantities in a market (“scale flexibility”), which have important consequences for the prediction of the effects of a supply shock on market prices [13–16]. A temporary or permanent reduction in catches may not only lower the costs, but also raise revenues, profits and resource rent depending on the proportional responsiveness of price to proportional changes in the quantities of the produce available i.e. the price flexibility for each product.

Recent spikes in TAC, and the corresponding rise in catch, of the Eastern Atlantic bluefin tuna (EABFT) stock may have seriously impacted the global bluefin tuna (BFT) price through the auction market in Japan, which generally acts as a global price-setter for bluefin sashimi trade. Previous estimates of price flexibility and inverse demand for tuna products have largely been *ad hoc* and focused on single species [17–19]. Without an empirical estimation of the theoretically plausible demand system, consumption substitution possibilities among species are excluded and estimates of the price flexibility could be biased [20–21]. Depending on the price responsiveness, increases in TAC of bluefin tuna can lead to price decreases at a greater proportion than the increase in quantity, and revenue and profit decrease shall soon follow.

Understanding this underlying market force is necessary if more fundamental management and conservation questions are to be answered. Is a stabilizing quota at a conservative level for global BFT a better option for the industry, and related industries? What was the impact of the recent spike in eastern Atlantic BFT catch on the price of Atlantic BFT and its substitutes? Would boosting the eastern Atlantic BFT quota further benefit the fishing industry? And who stands to win and lose from supply increases?

This study proposes to better understand the implications of these price effects through an original estimation based on the General Synthetic Inverse Demand Systems (GSIDS) approach [22]. This family of demand systems nests several flexible specifications and gives more robust estimates than other demand models [23–24]. The estimates of own- and cross- quantity flexibility and scale flexibility can be used to assess the impact of global quota management control and other supply shifters.

The paper is organized as follows: the marketing value chain of sashimi-grade tuna products is introduced in the second section to justify the estimation procedure motivated by the market delineation literature. The GSIDS model is presented in the third section, followed by presentation of the results. Elasticity and flexibility coefficients found in the empirical literature and their consequences on fisheries management are discussed in the last section.

### Global bluefin tuna landings and its auction market in Japan

**Fig 1:**
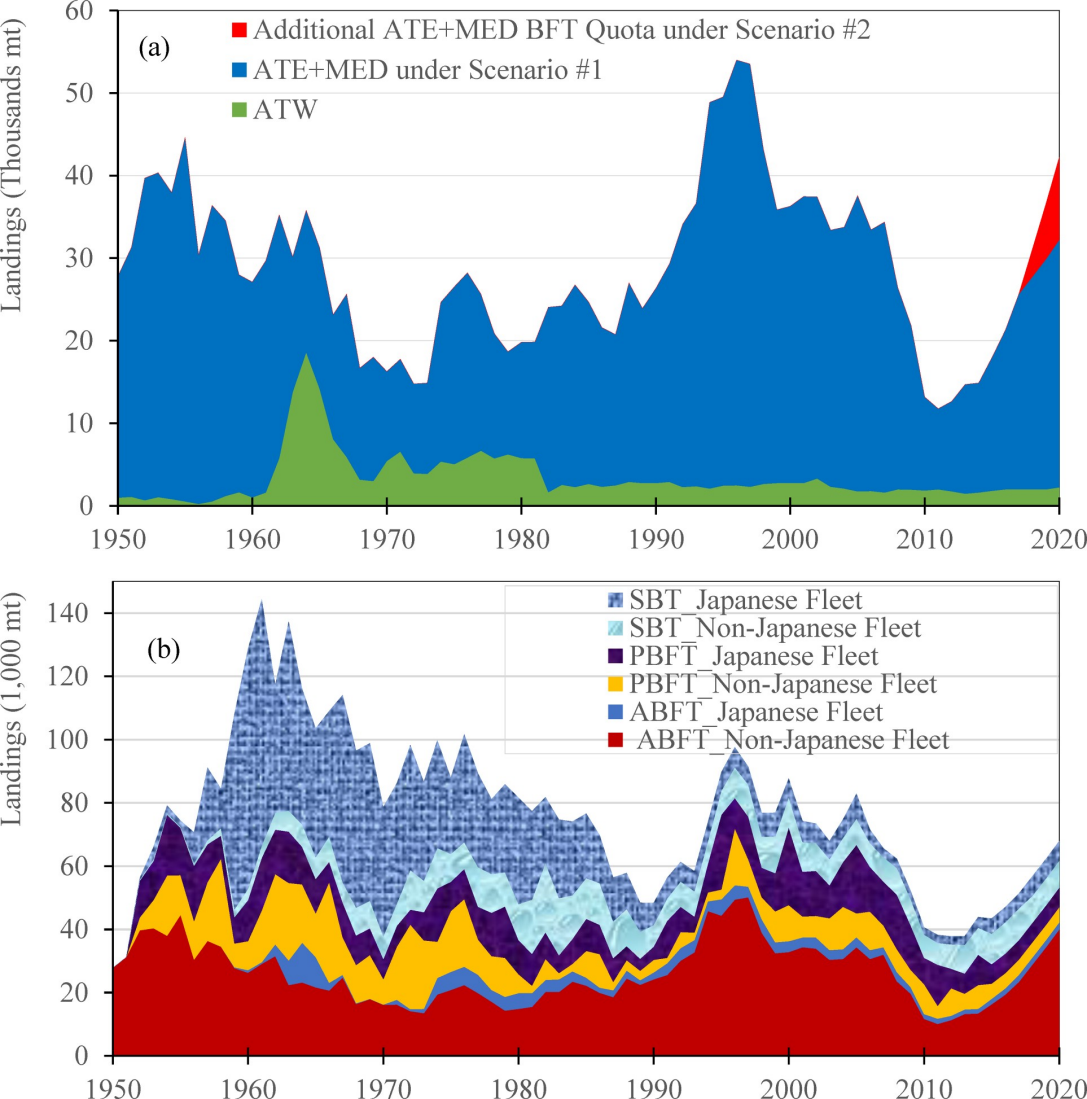
Cumulative annual supply of the bluefin tuna. (a) from Atlantic bluefin tuna by Stock Area (upper panel), with the abbreviation for Western Atlantic, Mediterranean, and Eastern Atlantic as ATW, MED, and ATE. (b) from global bluefin tuna by fishing Countries (lower panel).

Globally, there are three species of BFT: Atlantic (the largest and most threatened [25]), Pacific, and Southern BFT, which have all been regulated by TAC in their trade, including import, export, and re-export, since the 1990s. Since 2015, while both Pacific BFT and Southern BFT have shown no signs of increasing TACs, the eastern Atlantic BFT stock has started to show signs of rebuilding with a hike in TAC by 20 percent per year between 2015 and 2017 (a 75.22% increase over three years), while the TAC for western Atlantic BFT stayed the same at 2,000 metric tons (mt). As depicted in Fig 1, with the rise of eastern Atlantic BFT quota from 13,500 mt in 2014 to 23,655 mt in 2017, the global BFT landings increased by 16.92% from 43,895 mt in 2014 to 51,322 mt in 2017.

The stock area of the Atlantic bluefin tuna landings (Fig 1(A) from 1950–2016 is defined by IATTC as Western Atlantic, Mediterranean, and Eastern Atlantic and abbreviated as ATW, MED, and ATE. The projected TACs from 2017–2020 in Fig 1, defined by this study under Scenario #1, is to simulate if the quota for ATE+MED will increase to 30,000 mt in 2020, or will increase to 40,000 mt under Scenario #2.

The two populations of Atlantic BFT, the Eastern (EABFT) and Western (WABFT) stocks mix. The majority of the Atlantic BFT landings come from the eastern population (including catch in the Mediterranean Sea) (Fig 1(A)). Before 1950, fishermen in the western Atlantic held little interest in BFT. Commercial catch was virtually nonexistent, but demand soon quickly grew in the following years. In 1964, for example, approximately 18,000 mt of BFT were caught in the western Atlantic, which was 18 times larger than the 1960 catch level. This intense fishing pressure soon took its toll, leading to a dramatic decrease in the western Atlantic BFT population. By the end of 1990s, the catch had fallen by 80 percent. In 1998, recognizing that the population was at a low point, the International Commission for the Conservation of Atlantic Tunas (ICCAT) implemented a recovery plan that set the TAC, and instituted a stricter minimum legal size to limit fishing mortality of juvenile Atlantic BFT. In 2009, many governments and environmental organizations called for a suspension of international trade in BFT. ICCAT finally responded by reducing the TAC to scientifically recommended levels. In 2006, ICCAT (based on Recommendation Rec-06-05) adopted a 15-year rebuilding period for Eastern Atlantic and Mediterranean BFT, starting in 2007 and continuing through 2022, with the objective of recovering the stock to the biomass level that enables a fish stock to deliver the maximum sustainable yield (BMSY), with greater than 50% probability (increased to 60% probability in 2010).

Fig 1(B) shows that global landings of BFT reached 143,000 mt in 1961 and fell below 50,000 mt by the early 1990s—a 65% decrease. Landings subsequently increased to 80,000 mt but since 2010 fell again to lower than 40,000 mt. Because the FAO global tuna landings dataset is only updated through 2010, the landings from 2011 to 2015 are collected from reports provided by three tuna Regional Fishery Management Organizations (RFMOs): ICCAT, IATTC, and CCSBT. The projection of future landings is plotted based on possible TAC scenarios set by the RFMOs from 2016 to 2020.

For example, the range of the shocks was specified to simulate an increase in the quota of EABFT to 30,000 mt (scenario #1) or 40,000 mt (scenario #2) in 2020. Under EABFT quota scenario #1, annual global BFT landings are expected to be 57,917 mt in 2020–31.94% more than the global landings in 2014. Under EBFT quota scenario #2, the annual global BFT landings will reach 67,917 mt in 2020—a 54.73% increase from 2014.

According to the latest ICCAT stock assessment in 2014, the eastern Atlantic BFT population increased dramatically. The goal of achieving BMSY (through 2022), with at least 60% probability, might have already been, or will soon be reached. Following the advice of scientists and better controlling for overcapacity and Illegal, Unreported, and Unregulated (IUU) fishing has been effective. However, there is no discussion of how ICCAT should consider adding a new phase to the current recovery plan. Nonetheless, while eastern Atlantic BFT is on the road to recovery, ICCAT scientists have repeatedly pointed out a “problematic” level of uncertainty in the assessment for eastern Atlantic BFT, which makes it difficult to determine the speed and magnitude of the recovery. As indicated by previous ICCAT Standing Committee on Research and Statistics (SCRS) reports, a long-lived species such as bluefin tuna requires some time (over 10 years) for the stock to realize the benefit of recovery. The overhauled stock assessment, which was supposed to be completed in 2015 but was delayed until July 2017, was intended to address this uncertainty.

Fifteen governments hold shares of the eastern Atlantic BFT quota, with the EU nations holding 59% of the total eastern quota. Multiple countries determine the quota, including Spain, Italy, France, Japan, and N. African Countries. In addition, the EU industry has considerable influence in ICCAT’s TAC decision-making.

Although BFT have been part of the Mediterranean diet for thousands of years, the 1990s marked the explosion of the industrial-scale fishery in the eastern Atlantic. That decade brought a significant increase in the size of the purse-seine fleet—fishing boats that use large nets to surround the entire schools of bluefin. Rising Japanese demand for fatty tuna to accommodate the growing appetite for high-quality sushi also led to the development of tuna “ranching.” In 2015, purse-seiner fleets/countries with ranching capacity in the eastern Atlantic landed 63.39% of eastern Atlantic BFT. The gain in weight after 6 months of ranching was significant, for the same quota ranching operations effectively sent 30–60% more weight of bluefin tuna to Japan. In this case, the negative impact upon price from an increase in TAC for the purse-seining fleet targeting juvenile EABFT for ranching will be even more severe than when viewed strictly through the lens of quota allocations. Every additional unit of TAC used to capture juvenile BFT yielded 1.3 to1.6 times the volume when sold at auction, furthering the impact on the auction price. Ranching’s ability to deliver higher volume to the market than what was directly harvested means ranching operations had more impact on price than they otherwise would have. Note that none of the western Atlantic BFT was ranched, and the majority of those catches were shipped fresh directly to Japan for auction.

Bluefin is typically consumed as sashimi. Sashimi is defined as sliced raw fish meat served on a plate with various vegetables (Miyake *et al*. 2010, p. 63). The belly meat of bluefin tuna is the most demanded part for consumers. Japan constitutes the largest fresh, chilled, and frozen market for tuna sashimi in the world (60 to 80% of global demand). Since the early 90s, more than 80% of the fresh and frozen Pacific BFT, Atlantic BFT and Southern bluefin (SBT) sashimi consumption in Japan have come from imports, while the Japanese domestic landings of long-liners have steadily fallen (Fig 1(B)).

Most of tuna auctioned in Japan are measured in terms of Dressed Weight (DWT). DWT measures the weight of the animal after it has been gilled, gutted, and the head and fins have been removed. The corresponding round weight (which was reported in catch statistics) is 1.25 times the DWT, based on the ICCAT Conversion factors for fish products adopted by the SCRS for major species. In some cases, landings of tunas are reported in terms of Belly Meat (BM), which has a corresponding round weight are 10.28 times that of the BM.Imports of frozen SBT into Japan increased steadily from 1997–98 due to successful cage aquaculture in Australia, resulting in a downward trending SBT import price. The supermarket channels in Japan have attempted to attract customers by offering special discount for farmed SBT whose price is cheaper and supply more stable [26]. The domestic price has become more sensitive to imports. In general, BFT prices in Japan have displayed a decreasing trend since the economic recession of the 1990s (Miyake *et al*. 2010).

In addition, those frozen tuna consumed in Japan is called super frozen tuna, have been frozen at -60°C, and approximately 80% of tuna sold in the Japanese market lies in this category. This unique method of freezing tuna below -60°C on board maintains the freshness of the catch. Tuna is frozen before rigor mortis sets in. Immediately after the tuna is caught, it is quickly cleaned, bled, processed, and flash frozen to -60°C. This process prevents dehydration, spoilage, and bacteria growth—with very little moisture loss when thawed and no added chemicals or preservatives. It is said to be “fresher than fresh.”

Because of the scarcity of fresh BFT and extremely high prices in Japan, substituting different tuna species and alternating between fresh and frozen tuna is common [27–28] [20]. Japanese consumers have many substitutes to BFT, such as bigeye and yellowfin tuna. Bose and McIlgorm [20] utilized cointegration analysis to show that the substitution of yellowfin and bigeye tuna for bluefin tuna clearly exists in Japan. However, the price response to fluctuations in landings was not addressed because only price data were used in their analysis. Sun and Wang [29] investigated the monthly fresh tuna auction market structure and the relationship between major fresh tuna auction markets in Taiwan, and major fresh tuna auction markets in Tokyo. A multivariate ARMA model with a seasonal adjustment factor showed a significant relationship between Taiwanese and Japanese prices. Sun and Hsu [30] examined the price linkages of the frozen yellowfin and bigeye tuna markets for canning across three countries—Japan, Taiwan, and South Korea. Based on an error-correction model, they showed that all the price series exhibited similar linear long-run change patterns with the law of one price prevailing internationally. However, the price response relationship they obtained could be applied to the frozen tuna raw material market for canning and is not sufficient to address how the prices of bluefin tuna might be impacted by either bigeye or yellowfin tuna on the sashimi market.

With a multivariate Markov-switching error-correction model, Wu [31] demonstrated that the import prices of frozen bigeye and yellowfin tuna for the sashimi market in Japan had no significant impact on the decreasing price trend of the same species caught by the Japanese fleet during1990-2006. They instead found the Japanese auction price demonstrated a structural change after the introduction of comparatively less expensive farmed Bluefin tuna to Japanese grocery markets in the late 1990s.

Chiang *et al*. [21], examining the role of inventories on tuna auction prices in Japan and using the Rotterdam inverse demand system, found that frozen tunas of different species (bluefin, bigeye and yellowfin tuna) were likely to be close substitutes in consumption. Fresh and frozen tunas of the same species also demonstrated substitution in consumption, and inventories of frozen tunas had significant impacts on auction prices.

Consumer preference for sashimi products appeared to have changed in response to the Asian financial crisis in 1997 and 1998. After this breakpoint, Japanese demand shifted towards cheaper products, such as frozen yellowfin and bigeye, instead of the more expensive fresh/chilled bluefin tuna species [9]. Since a large majority of the premier sashimi-grade tuna is shipped daily from all over the world to the Tsukiji Market in Tokyo, the central metropolitan wholesale fish market in Tokyo, the BFT auction prices observed in Japan capture the tuna price response to global supply changes. Most of the BFT auctioned in the Tsukiji Market were fresh. In contrast, the majority of the frozen BFT sales in Japan were directly sold to buyers without entering the auction market. The BFT auction price in the Tsukiji Market significantly drives the direct sale pricing because of the close substitution between fresh and frozen BFTs. The Tsukiji Market thus creates a reference price for both fresh and frozen BFTs within Japan and globally.

The frequent adjustments of the monthly bluefin auction price in Tsukiji demonstrate high responsiveness to the highly seasonal global landings. This justifies the use of an inverse demand analysis, also advocated by other analysts of global seafood markets [32] [16].

Estimates of the responsiveness of price to landings would help inform policy analysis on the economic benefit of global quota management control and the impact of changes in fishing capacity upon the value of total landings. Success of quota control relies crucially on guaranteed higher profit when the quota is managed such that the net present value of the fishery resources is maximized in the long run [33].

## A general synthetic inverse demand system approach

### Inverse demand system

In a study of the price formation of fish, Barten and Bettendorf (1989) developed a Hicksian inverse demand model, known as the Rotterdam inverse demand system (RIDS), using the direct utility function and the Hotelling-Wold identity. Barten [34] compared the RIDS and the almost ideal inverse demand system (AIIDS), along with two mixed models—one with Rotterdam-type price effects and AIDS-type income effects and the other with AIDS-type price effects and Rotterdam-type income effects. Barten [34] proposed a synthetic direct model that combined the features of the latter four models and allowed non-nested hypothesis tests among models. Brown *et al*. [22] specified a family of the general synthetic inverse demand systems (GSIDS), which included two flexible specifications: the RIDS and the AIIDS on the one hand [24], and the inverse demand system proposed by Laitinen and Theil [23] with a fourth variant on the other.

### Background: Inverse demand systems

The question underlying inverse demand approaches is: on a market with several exchanged commodities, how relative variation of prices depend on relative variation of landings quantities. Barten and Bettendorf [24] approach assumes there are n goods, denoted i, j, k, a price vector P = (*p*_*i*_) ∈ $R_{+}^{n}$, a quantity vector Q = (*q*_*i*_) ∈ $R_{+}^{n}$, and a maximum expenditure m ∈ $R_{+}^{n}$. The solution of maximization a utility function U(Q) subject to a budget constraint ∑_*i*_*p*_*i*_*q*_*i*_ = m and express *π*_*i*_ = *p*_*i*_/m as an explicit function of Q, P, and gradient of U: *D*_*i*_ = ∂U/∂*q*_*i*_.

$${\pi_{i}=D}_{i}/\sum_{j}q_{j}D_{j}$$ (1)

The log derivatives of Π = (*π*_*i*_) can be expressed as functions of log derivatives of Q, of partial derivatives of U and of its Hessian: *H*_*ij*_ = ∂^2^*U*/∂*q*_*i*_∂*q*_*j*_. That is,

$$\frac{d\pi_{i}}{\pi_{i}}=F(\frac{dq_{i}}{q_{i}},D_{j},H_{jk})$$ (2)

Barten and Bettendorf proceed as follows. The mathematical expression of U is unknown. Putting *w*_*i*_ = *π*_*i*_*q*_*i*_, they get the expression:

$$w_{i}\frac{d\pi_{i}}{\pi_{i}}=h_{i}\sum_{j}w_{j}\frac{dq_{j}}{q_{j}}+\sum_{j}h_{ij}\frac{dq_{j}}{q_{j}}$$ (3)

Where *h*_*i*_ and *h*_*ij*_ are, as well as F, complicated expressions in terms of the gradient and the Hessian of U. *h*_*i*_ and *h*_*ij*_ are considered as characteristics of the inverse demand system: *h*_*i*_ is the scale flexibility, and *h*_*ij*_ the compensated cross-price flexibility. Having time series of prices *p*_*i*,*t*_ of quantities *q*_*i*,*t*_ of expenditure *m*_*t*_, then the each variable can be expressed as, *dq*_*i*,*t*_ = *q*_*i*,*t*_—*q*_*i*,*t*−1_, *π*_*i*,*t*_ = *p*_*i*,*t*_/*m*_*t*_, and *dπ*_*i*,*t*_ = *π*_*i*,*t*_—*π*_*i*,*t*−1_. The unknown parameters *h*_*i*_ and *h*_*ij*_ are then estimated with a maximum likelihood method.

To express different ideas of flexibility, Brown *et al*.[22], Holt [35] and Sun *et al*. [33][36] considerations leads to an alternative formulation by introducing new parameters *d*_1_ and *d*_2_.

$$w_{i}\frac{d\pi_{i}}{\pi_{i}}=(h_{i}-d_{1}w_{i})\sum_{j}w_{j}\frac{dq_{j}}{q_{j}}+\sum_{j}{(h}_{ij}-d_{2}w_{i}(\delta_{ij}-w_{j}))\frac{dq_{j}}{q_{j}}$$ (4)

The constraints on these parameters will lead to several type of models. The estimated parameters and models can be given several economic interpretations. The analysis of different inverse demand systems here can be viewed as allowing the scale flexibility, and the compensated cross-quantity flexibility to be variational parameters dependent on budget shares [22]. The model specification of General Synthetic Inverse Demand System (GSIDS) is shown in S1 Appendix.

The demand system itself is subject to several *ex-ante* decisions on the part of the analyst concerning the appropriate market delineation (the limits of the relevant market). Several recent studies have shown strong globalization of the tuna markets [37–40] [33] and identified two separate market chains: purse-seine/cannery-grade and long-line/sashimi-grade tuna markets [41] [42] [9] [36]. Each of the two distinct markets, purse-seine/cannery-grade and long-line/sashimi-grade, are highly integrated at the global level by both price and commodity flows across locations and species, making any regional change in catches important to the entire industry. The concentration of processors and traders is high, and the information is rapidly transmitted from one location to the other [43–44], with the leading sashimi-grade tuna market located in Japan. In response to these market dynamics, a set of demand equations including different species and products would be needed for sashimi-grade tuna (fresh and frozen bluefin, southern bluefin, and bigeye tuna) on the Japanese market.

### Data collection and inverse demand system parameterization

**Fig 2:**
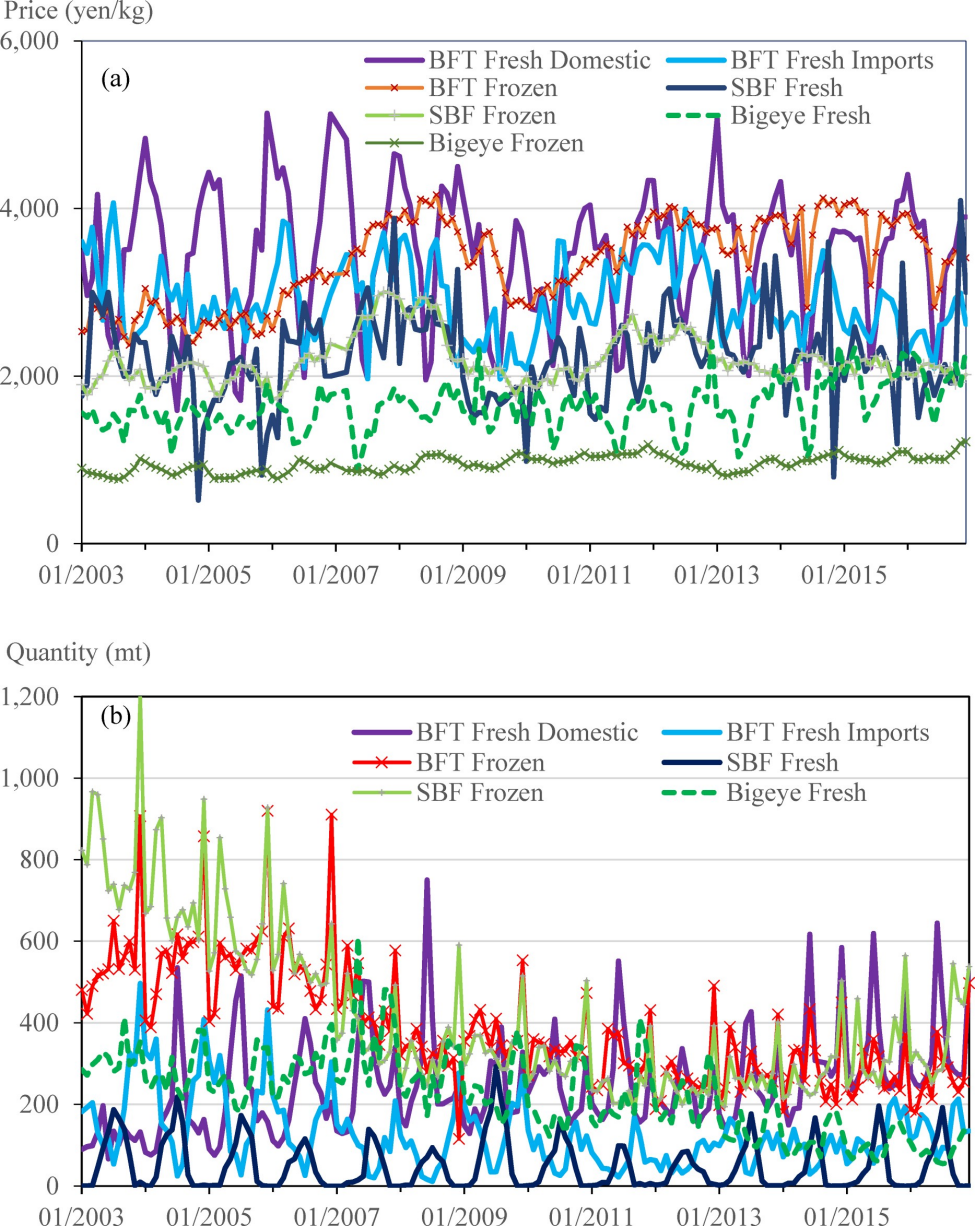
Monthly auction market of bluefin and bigeye tuna at Tokyo, Japan (January 2003 to December 2016). (a) Auction price (Nominal price without inflation adjustment). (b) Auction quantity.

All of the data used in the model are compiled from the monthly average tuna auction price and cumulative monthly quantity from January 2003 to December 2016, with more than three transactions across more than three dealers and sellers at Tokyo Metropolitan Central Wholesale Market (Abbreviated as “Tokyo Market”, http://www.shijou.metro.tokyo.jp/), and cannot be de-identified for any individual information. Those transactions at Tokyo Market represent not only transactions at the Tsukiji central market but also at the Adachi and Ota markets. The monthly total value for sashimi-grade tuna auctioned at the Tokyo Market was about 6–8 billion yen during 2003 to 2016, and accounts for 80% of the global bluefin tuna sashimi consumption. Although frozen bigeye tuna (BET) accounts for more than half of the volume auctioned, fresh and frozen BFT accounts for more than 60% of the monthly auction value. Fig 2(A) shows that fresh BFT demonstrates the highest price at the auction with a strong seasonal pattern. In comparison, the auction price of frozen bigeye tuna remains the lowest and is unresponsive to any change in BFT supply or seasonal variation in BFT prices. These trends reflect the relatively higher level of demand for BFT than bigeye and yellowfin in addition to the higher value typically achieved by fresh tuna when compared to frozen tuna.

To capture how the BFT price responds to the volatility of global supply BFT shock, such as indicated in Fig 2(B), six species/product forms are included in the inverse demand system. Table 1 shows that the fresh BFT supplied by Japanese fleet and non-Japanese fleets, including supplies from both Pacific and Atlantic BFT, accounts for 22.25% and 9.31% of the total sale revenue at the Tokyo Market in Japan, between January 2003 and December 2016. Frozen BFT, including supplies from both Pacific and Atlantic BFT, accounts for 33.29% of the total revenue share. The total aggregated revenues from BFT accounts for 64.85%, and the southern BFT and bigeye tuna accounts for the 25.91%, and 9.24%, respectively, of the total sales revenue. Variability of budget shares is also much more pronounced for bluefin than bigeye tuna.

**Table 1:** Monthly sample statistics of tuna auction market in Tokyo, Japan.

	Mean	Std. Dev.	Minimum	Maximum
**Quantity Sold (metric tons)**				
BFT_Fresh_Domestic (Japanese Fleet)	250	123	67	750
BFT_Fresh_Import (Non-Japanese Fleet)	127	86	11	496
BFT_Frozen	389	147	115	920
SBT_Fresh	49	60	0	292
SBT_Frozen	418	203	185	1207
Bigeye_Fresh	222	100	55	609
**Nominal Average Auction Price (yen/kg)**				
BFT_Fresh_Domestic (Japanese Fleet)	3,457	775	1,590	5,137
BFT_Fresh_Import (Non-Japanese Fleet)	2,905	460	1,965	4,067
BFT_Frozen	3,366	507	2,369	4,162
SBT_Fresh	2,228	536	519	4,095
SBT_Frozen	2,194	285	1,723	3,004
Bigeye_Fresh	1,636	296	869	2,407
**Revenue Share**				
BFT_Fresh_Domestic (Japanese Fleet)	22.25%	8.32%	3.70%	33.14%
BFT_Fresh_Import (Non-Japanese Fleet)	9.31%	4.83%	1.05%	22.35%
BFT_Frozen	33.29%	4.38%	10.61%	44.56%
SBT_Fresh	3.02%	3.59%	0.00%	14.50%
SBT_Frozen	22.90%	5.43%	15.01%	38.62%
Bigeye_Fresh	9.24%	3.29%	2.44%	19.10%

Source: Monthly Statistic Report of Tokyo Metropolitan Central Wholesale Market, http://www.shijou.metro.tokyo.jp/torihiki/geppo/.

**Fig 3:**
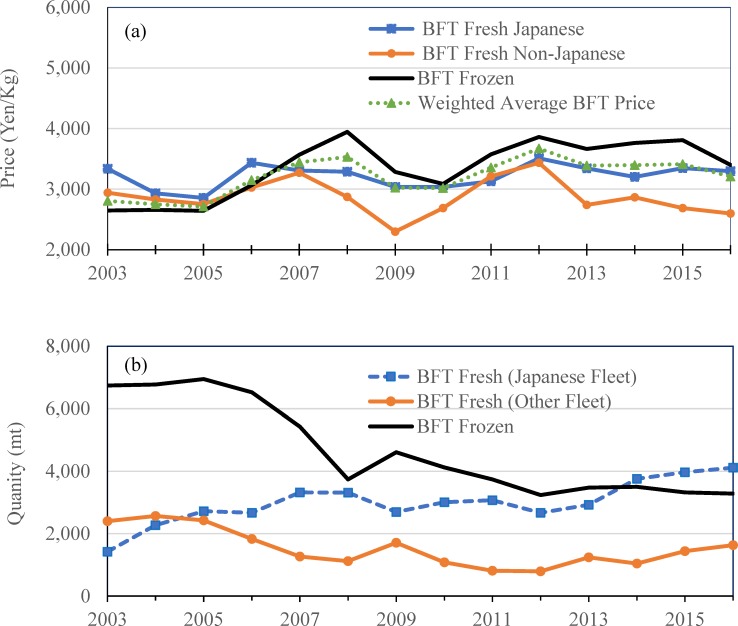
Annual auction market of bluefin tuna at Tokyo, Japan. (a) Monthly auction quantity weighted average price (Nominal price without inflation adjustment). (b) Auction quantity.

Fig 3(A) show that the annual weighted average nominal auction prices for fresh bluefin supplied by non-Japanese fleets from either the Pacific or Atlantic Oceans has trended down from 3,434 yen/kg in 2012 to 2,864 yen/kg in 2014 and further falling to 2,598 yen/kg in 2016. The arithmetic weighted average price of all species in time *t* can be written: ${\bar{p}}_{t}=\sum_{i}{SH}_{it}p_{it}$ where *SH*_*it*_ denotes the revenue share of species *i* in time *t* (revenue of species *i* divided by revenue of all species in time *t*) and *p*_*it*_ denotes the price of species *i* in time *t*.

This represents a total decline of 24.35% in price from 2012 to 2016, while global supply more than doubled from 792 mt in 2012 to 1,632 mt in 2016—an increase in landings attributable to the spike in catch/quota of the eastern Atlantic BFT since 2014. In addition, the weighted average auction prices across all fresh and frozen Pacific and Atlantic BFT, shown in Fig 3(A), also exhibit downward trends since 2012.

In a GSIDS estimation, supply at the seafood auction market is treated as fixed in the short run, with the prices at the auction adjusting to a point that clears the market. (In other words, an equilibrium price is reached where the quantity supplied–in this case, the entirety present at the market–and demanded are exactly equal.) Because frozen products often do not go through the Tokyo Market, the auction market dataset represents most of the fresh product supply. Almost all of the frozen bluefin tuna products are not auctioned at Tokyo Market and was supplied by most of the ranching bluefin tuna (instead of aquaculture–to date, bluefin aquaculture is not economically viable). In ranching, live juvenile bluefin tuna are corralled into pens and fattened up for a minimum of 6 months, before shipment to Japan for auction, where it is mixed with “wild caught” (in this case, referring to those bluefin that are caught and immediately shipped to auction). Importantly, because the juveniles caught for ranching still count against the quotas and ICCAT sets the quotas for bluefin tuna, the supply of fresh bluefin tuna in the auction market is treated as exogenous.

## Results of the inverse demand analysis

The estimated GSIDS gives the price responses of six tuna products, summarized in Table 1, to quantify changes in monthly totals at the tuna auction market in Tokyo. Additional supporting information section containing the TSP program codes are provided in the public repository RePEc (Research Papers in Economics) at the IDEAS working paper series no. 1901 (https://ideas.repec.org/p/nto/wpaper/1901.html) archived by Institute of Applied Economics, National Taiwan Ocean University.

The GSIDS approach is chosen because it overcomes the usual problems of specification by covering a broad range of inverse demand systems (IDS) and offering the best tradeoff between a good fit of observations and a theoretically grounded approach. First differences of the nominal prices time series are taken because differentials must be approximated to first differences for the differential inverse demand system to be estimated.

There are different assumptions for the demand parameters. The RIDS approach assumes fixed demand parameters (i.e. scale and cross-quantity or Antonelli coefficients) and the AIIDS assumes variable demand parameters where scale and cross-quantity coefficients are a function of budget shares. A set of 7 synthetic models and restricted versions of IDS are estimated, and Table 2 shows the likelihood-ratio (LR) test result for each of the models. Based on the LR test, the synthetic IDS model best fits our data, with a higher log likelihood value than all other IDSs.

**Table 2:** Maximum likelihood test statistics of inverse demand systems of the tuna sashimi market in Tokyo, Japan.

System	d1		d2		Log Likelihood Value (LLV)	Likelihood-Ratio (LR) Test ^a^		
Synthetic	1.116	***	0.133	***	2532.30			
	(0.071)^b^		(0.021) ^b^					
RIDS	0.000		0.000		2423.53	217.54	***	(2)
Laitinen-Theil (CBS)	1.000		0.000		2515.96	32.68	***	(2)
AIIDS	1.000		1.000		2255.97	552.66	***	(2)
RAIIDS (NBR)	0.000		1.000		2223.07	618.46	***	(2)
free d1, zero d2	1.127	***	0.000		2517.44	29.72	***	(1)
	(0.072) ^b^							
free d2, zero d1	0.000		0.1725	***	2438.87	186.86	***	(1)
			(0.026) ^b^					

^a^ The LR test value = 2*(LLV for each model—LLV for the synthetic); the degrees of freedom in parentheses.

^b^ Numbers in parentheses are standard errors of parameter estimates.

*** for 1% level of significance, ** for 5%, and * for 10%

### Scale flexibility

Since the synthetic IDS, best fits our data, all forthcoming estimates (scale flexibility, cross flexibilities) come from this model. The estimated scale flexibilities of all products under the “Scale Flexibility” column in Table 3 all have the expected negative sign and are significantly different from zero at the 1% level. In reference to their standard errors, neither of the scale flexibility coefficients for fresh imported bluefin (-1.004) and fresh southern bluefin (-0.899) tunas are significantly different from unity in absolute value.

**Table 3:** The scale, uncompensated own-quantity flexibility and uncompensated cross-quantity flexibility of the tuna sashimi market in Tokyo, Japan.

	Scale Flexibility		Uncompensated Price Flexibility											
			BFT_Fresh Japanese Fleet		BFT_Fresh Non-Japanese Fleet		BFT Frozen		SBT Fresh				SBT Frozen		Bigeye Fresh	
BFT_Fresh Japanese	-1.152	***	-0.478	***	-0.103	***	-0.319	***	-0.029	***	-0.141	***	-0.081	***
	(0.044)		(0.018)		(0.009)		(0.017)		(0.003)		(0.016)		(0.012)	
BFT_Fresh Non-Japanese	-1.004	***	-0.219	***	-0.219	***	-0.281	***	-0.029	***	-0.192	***	-0.064	***
	(0.082)		(0.027)		(0.023)		(0.031)		(0.006)		(0.028)		(0.021)	
BFT Frozen	-0.911	***	-0.159	***	-0.068	***	-0.375	***	-0.022	***	-0.215	***	-0.072	***
	(0.034)		(0.012)		(0.008)		(0.015)		(0.003)		(0.013)		(0.010)	
SBT_Fresh	-0.899	***	-0.163	***	-0.032		-0.249	***	-0.124	***	-0.163	***	-0.121	***
	(0.136)		(0.038)		(0.077)		(0.049)		(0.019)		(0.037)		(0.025)	
SBT_Frozen	-0.900	***	-0.084	***	-0.069	***	-0.318	***	-0.022	***	-0.405	***	-0.003	
	(0.036)		(0.014)		(0.009)		(0.014)		(0.003)		(0.022)		(0.014)	
Bigeye Fresh	-1.224	***	-0.210	***	-0.083	***	-0.365	***	-0.048	***	-0.079	***	-0.439	***
	(0.090)		(0.021)		(0.008)		(0.031)		(0.003)		(0.021)		(0.040)	

Note: Asymptotic standard errors in parentheses.

*** for 1% level of significance, ** for 5%, and * for 10%.

A summary of the scale flexibilities for all products is shown in Table 4. We now turn to the implications of the scale flexibilities upon Tokyo Market prices, producer revenues, and operating profits for each product and discuss whether the products are necessary or luxury goods.

**Table 4:** The impact of a greater global supply of bluefin and bigeye tuna on the price of each product.

Product	Scale Flexibility (Absolute Value)	Increasing Global Supply by 1% Leads to a Decline in Price by	Resulting Total Revenue Change
Atlantic & Pacific bluefin, fresh (Japanese fleet)	1.15	1.15%^a^	Decrease
Atlantic & Pacific bluefin, fresh (Non-Japanese fleet)	1.00^c^	1.00%^c^	Constant
Atlantic & Pacific bluefin, frozen	0.91	0.91%^b^	Increase
Southern bluefin, fresh	0.90^c^	0.90% ^c^	Constant
Southern Bluefin, frozen	0.90	0.90%^b^	Increase
Bigeye, fresh	1.22	1.22%^a^	Decrease

^a^ Declines in price greater than 1%, such as 1.15% for Product #1, mean that price drops are larger than the supply increases, resulting in reduced total revenue by 0.15% if the supply can increase by 1% of EBFT landings for Japanese fleet. However, if Japanese fleet didn’t increase their EBFT landings, their revenue will decrease the same percentage as the decline in price.

^b^ Declines between 0 and 1% mean price drops are less than global supply increases, resulting in more revenue. However, this result is only possible for the EABFT industry, since their supply could increase during 2015–2017 and possibly further increase during 2018–2020.

^c^ Scale flexibility not significantly different than one.

Scale flexibilities are greater than unity (in absolute values) for both Atlantic and Pacific BFT from the Japanese fleet (-1.15) and fresh bigeye tuna (-1.22), and thereby are scale flexible. Prices would decrease more rapidly than increases in aggregate supply and TACs for all products, so that producer revenues and profits would fall, with profit losses further increased by increases in operating costs. Conversely conservation through lower aggregate supply and lower TACs would raise revenues and profits (boosted by lower expected operating costs). These two fresh products are necessities in the Tokyo Market (∞<*f*_*i*_<−1).

The scale flexibilities are not significantly different from unity for fresh Atlantic and Pacific BFT (-1.00) not from the Japanese fleet or fresh southern BFT (-0.90). These unitary own-quantity price flexibilities imply that prices will increase (decrease) by1% if total supply of all products decreases (increases) by 1% for each. Total sales revenue will remain constant for different catch levels, under the assumption that the worldwide demand for BFT is fixed in the short to intermediate run, although profits would fall (rise) due to lower (higher) expected operating costs. Moreover, the sales shares are constant, and consumption of these two products is independent of the level of total expenditure.

Under the SSP (Shared-Socioeconomic Pathways) scenarios developed by Intergovernmental Panel on Climate Change (IPCC), the BFT prices might be impacted by a steady trend of some possible futures at the global scale. For example, such as the SSP5 scenario (rapid growth in the long run), characterized by a global economic growth rate of 3.5% per year up to 2040 [45], is not considered in this study. The unitary scale elasticity indicates that preference for fresh imported bluefin and fresh southern bluefin tunas is homothetic, which means that the sales shares are constant and that consumption of these two products is independent of the level of total expenditure [24]. Moreover, at the margin, normalized price is proportional to marginal utility. Therefore, as consumption of all goods increases by l%, the marginal utility of necessities declines more than proportionately (∞<*f*_*i*_<−1) and the marginal utility of luxuries declines less than proportionately (−1<*f*_*i*_<0).

Scale flexibilities are less than unity for both frozen BFT (-0.91) and frozen southern BFT (-0.90), and thereby scale inflexible. Prices would decrease proportionately less than proportionate increases in aggregate supply, so that producer revenues for these two frozen products would climb. Whether profits rose or fell would depend upon whether revenue increases outpaced expected cost increases. Conversely, conservation through lower aggregate supply and lower TAC for all products would raise revenues and profits from lower expected operating costs. Japanese consumers are still willing to pay a premium for sashimi-grade frozen BFT, since it is a luxury good (−1<*f*_*i*_<0).

Prices for the two frozen products are less responsive to changes in aggregate supply than the four fresh products or conversely, the prices for fresh products are more responsive than the prices for frozen products when aggregate supply changes (scale flexibilities for frozen products are smaller in absolute value than for fresh products). This stance is perhaps influenced by the frozen BFT’s significantly longer shelf life than fresh BFT and bigeye and the related capability of storing inventories and releasing when profitability or to keep markets supplied.

The revenue and profit impacts of increases in aggregate supply and TACs for all products from across the globe, or conversely declines in both, are unevenly spread among fisheries and product forms. Some gain while others lose or remain the same.

Because of the greater than unity scale elasticities for fresh BFT supplied by the Japanese fleet and for bigeye tunas supplied by all fleets, an increase in the TACs of global BFT would cause a decline in gross revenue for fishermen currently targeting BFT. In addition, even the fresh BFT supplied by other fleets shows a unitary scale flexibility, i.e., the total revenue would remain the same when total supply increases, but the fishermen would still possibly incur a loss since the increase in supply may come at higher operating expenses. More importantly, there is a negative spillover effect to fishermen operating in the Pacific and western Atlantic Oceans when only those operating in the eastern Atlantic increase their landings, because the decrease in the auction price would lead to a reduction in revenue for Pacific and Western Atlantic fishermen without an parallel chance to increase landings to overcome the decrease in price.

For three of the five products examined, prices would decrease more rapidly than aggregate supply increases, meaning that the revenue of the fishermen supplying these products would decline even if their landings increased when global supply of all species and product forms increases. This poses even more of a problem for western Atlantic bluefin fishermen, whose quotas stabilized as eastern Atlantic bluefin quotas rose annually by 20 percent from 2015 to 2017, and for Pacific BFT and fresh/frozen SBT fishermen, whose quotas declined or stayed the same when eastern bluefin quotas increased. Conversely, fishermen’s revenue would increase proportionately more than any proportionate reductions in supply, creating incentives for conservation. Profits would likely increase as well due to likely lower operating costs with the reduced fishing, boosting the conservation incentives.

The only sector not expected to be harmed by a greater aggregate tuna supply from all parts of the globe is the frozen Atlantic industry. For example, if the ICCAT quota increases, the fishermen catching Bluefin in the Pacific will see the price fall without receiving any of the extra quota. However, there are still many factors that could minimize an individual fisherman’s ability to profit from this circumstance and it depends on whether or not the individual fishermen-level action would be consistent with their country-level, and RFMO-level actions. Unless each country implements individually assigned quotas, for example, fishermen selling these products are not guaranteed to increase their own catch, even if their own fishery’s quotas are raised. Additionally, if quotas are capped, as they were for SBT in 2016 and 2017, fishermen could simply face lower prices as they not be able to boost their individual catch as aggregate supply increases.

### Own- and cross-quantity price flexibility

The uncompensated own-quantity price flexibilities of demand (the shaded diagonal elements of Column 3) are significantly negative, smaller than the corresponding scale flexibility (in absolute value) for all products and are significantly different from zero at the 5% level of significance.

The uncompensated own-price flexibilities capture the combined effect of compensate price and scale flexibilities, i.e. they account for both the price and scale (expansion in the same proportion) effects. The uncompensated cross-price flexibilities (off-diagonal elements) are all q-substitutes and statistically significant. The q-substitution means that with a price rise, consumers in the Tsukiji market substitute away from the product to another. The q-substitution counters the impact of the product’s scale flexibility, leading to the uncompensated own-price flexibilities (capturing the combined effect of compensated price and scale flexibilities) smaller in value than the scale flexibilities.

These own-quantity price flexibility estimates are all less than unity (i.e. they are inelastic), implying that own prices are inflexible to changes in their own consumption, i.e. own prices demonstrate responses proportionately smaller than own quantity changes (allowing for both changes in the scale of consumption and responses to price in consumption of the commodity bundle). These inelastic own-quantity flexibilities create weak producer incentives to individually reduce wild or farmed bluefin tuna supply in each region because a 1% fall in supply leads to a less than 1% decline in price and hence a decline in total revenue from the product. These inelastic own-quantity flexibilities also imply that the corresponding price elasticities of demand are elastic, where sashimi-grade BFT tuna is commonly recognized as luxury good for which demand increases more than proportionally as income rises.

The uncompensated own-quantity price flexibilities of the fresh BFT supplied by domestic Japanese fleets (-0.478) and fresh bigeye tuna (-0.439), while inflexible, are larger than the own-quantity price flexibilities of the other products. The results suggest that prices for these products will fall more than the rest of the commodities with an increase in own supply, implying 0.478% and 0.439% reductions in marginal values with a 1% increase in own supply.

Tokunaga [46] also utilized Tsukiji market data, in this instance directly estimating an ordinary demand equation along with various supply shocks as instrument variables, and showed own-price elasticities of demand all greater than unity (in absolute value), also implying an elastic demand of bluefin tuna. However, Huang [47] argued that using directly inverted elasticities to represent flexibilities, or vice versa, may commit sizable measurement errors, and only elasticity from directly estimated ordinary demand systems should be used to evaluate the quantity effects of price changes. Note that Tokunaga [46] stated that it is beyond the scope of their paper to identify the substitutability across various bluefin tuna (and other tuna species) products, which is an advantage of the approach employed here.

Reciprocals of the price flexibilities provide a lower bound on the *p*-elasticities of substitution between products in direct demand by Japanese buyers in the Tokyo Market [48]. The *p*-elasticities of substitution measure the responsiveness in quantity directly demanded of a product *q*_*it*_ to a change in either that product’s own price *p*_*it*_, giving the own-price elasticity of direct demand ∂*lnq*_*it*_/∂*lnp*_*it*_, or to the change in the price of another product, *p*_*jt*_, giving the cross-price elasticity of direct demand ∂*lnq*_*it*_/∂*lnp*_*jt*_. ∂*lnq*_*it*_/∂*lnp*_*jt*_ > 0 for *p*-substitutes, so that as the price *p*_*jt*_ of the substitute product *q*_*jt*_ increases, the quantity directly demanded of product *q*_*it*_ increases. ∂*lnq*_*it*_/∂*lnp*_*jt*_ < 0 for *p*-complements, so that as the price *p*_*jt*_ of the complement product *q*_*jt*_ increases, the quantity directly demanded of product *q*_*it*_ decreases. *P-*elasticities correspond to direct demand, in which quantity demanded depends upon price, and *q*-elasticities correspond to inverse demand, in which price depends upon quantity supplied.

Since the reciprocal of the price flexibility forms the lower limit, in absolute terms, of the price elasticity [48], the difference of the true price elasticity of direct demand from the reciprocal of the price flexibility from inverse demand depends on the entire matrix characterized by the substitution and complementarity of price flexibilities with other commodities [47].

Table 5 provides these reciprocals, taken by inverting the matrix in Table 4. Because these are lower bounds, the *p*-elasticities should be as least as large as indicated by Table 5. Own-price direct demand is very elastic, indicating considerable responsiveness in quantity demanded of a product to changes in its own price. Pervasive positive cross-price price flexibilities and the q-complementarity found in Table 4 indicate *p*-substitution with direct demand, which at first glance is largely confirmed by the widespread positive and often large signs in Table 5. The pervasive *p*-substitutability among the different products is also highly elastic in most instances, as indicated by the large absolute values. Thus, buyers (“demanders”) in the Tokyo Market seem to easily substitute one product for another when there are relative price changes.

**Table 5:** Reciprocals of price flexibilities.

	BFT Fresh Japanese Fleet	BFT fresh Non-Japanese fleet	BFT Frozen	SBT Fresh	SBT Frozen	Bigeye Fresh
BFT Fresh Japanese fleet	-3.36	0.98	2.67	0.23	-0.80	-0.02
BFT Fresh Non-Japanese fleet	2.00	-6.56	1.56	0.47	1.36	0.19
BFT Frozen	1.47	0.36	-7.98	0.02	3.36	0.96
SBT Fresh	2.22	-1.80	1.21	-9.80	2.92	2.33
SBT Frozen	-0.92	0.72	5.35	0.38	-5.32	-0.88
Bigeye Fresh	-0.07	0.54	3.97	0.78	-2.03	-3.20

Exceptions to pervasive *p*-substitutability, however, start to emerge upon closer examination. One exception is fresh BFT from the Japanese fleet, in which the *p*-elasticities are small in absolute value, and there are even complementary products (given by negative signs). These results indicate that buyers in the Tokyo Market distinguish fresh BFT from the Japanese fleet compared to other products, i.e. the other products are not considered to be close *p*-substitutes. Another exception is frozen products that do not readily substitute in buyer direct demand for some fresh products as indicated by *p*-elasticities with positive signs that are small in absolute value and that are complements with other products as indicated by *p*-elasticities with negative signs. Thus *p*-substitution in direct demand is more concentrated upon fresh SBT and non-Japanese BFT. Frozen products (SBT and BFT) are highly substitutable for each other in Tokyo Market buyer direct demand (reciprocal values of 5.35 and 3.36 in Table 5) when there are changes in the relative prices of the frozen products. Tokyo Market buyers seem largely indifferent to the source of frozen products, i.e. frozen products are not differentiated products in the eyes of buyers.

The Morishima elasticities of complementarity (MEC), reported in Table A1, shown in S1 Appendix, are all positive, indicating pervasive q-complementarity and consistent with the widespread *p*-substitutability from Table 5. All values are inelastic, indicating that demand price ratios change proportionately less than the change in one of the quantities due to high substitution. The market for all three species of BFT is integrated and is considered highly *q*-substitutable between different product forms (fresh/frozen and Japanese/non-Japanese) and species of BFT (indicated by small and positive MECs). Nonetheless, the column and row MECs between the Japanese and non-Japanese BFT products are generally larger than the column and row MECs for the other BFT products (but not for fresh bigeye). The implications of demand system scale flexibilities on market behavior and incentives are important for fishery management and market incentives and should be taken into greater consideration by local regulatory bodies.

## Simulation of the impact of increasing the TAC of EABFT

This section examines the fishery for Atlantic BFT in greater detail. This fishery is complicated by the presence of two different stocks (that mix); the number of members of ICAAT, and the many States comprising the European Union; and the different types of fishing gear used to catch BFT, ranging from coastal longliners to high seas longliners to purse seiners. As shown in Fig 1(B), global BFT landings increased by 16.92%, from 43,895 mt in 2014 to 51,322 mt in 2017. This is due to an increase in landings of EABFT by 75.22% between the years 2015 and 2017.

EABFT’s TAC was renegotiated by ICCAT in November 2017 and set for the following three years, with a TAC of 28,200 mt for 2018, 32,240 mt for 2019, and 36,000 mt for 2020 [49]. Prior to the meeting, discussion suggested the TAC could even increase up to 40,000 mt, for example that Spanish industry group Balfego has been calling for an increase to 40,000 mt, approaching the catch levels when the stock was becoming severely depleted during 1990s. With bluefin landings elsewhere in the world expected to remain essentially unchanged, the increased landings of EABFT will push the global landings of BFT up and are, therefore, expected to lower the BFT, SBT and BET auction price in Japan. We now project the impact on the BFT auction price in Tokyo Market of these increases in EABFT TAC, based on the GSIDS demand system estimated in this study.

### Observed significant impact of the recent spike in catch of the EABFT on global price

Fig 3 show annual BFT price fluctuation in the Japanese auction market between the years 2003 and 2016 and demonstrate the significant impact of the recent spike in catch (or TAC; the two can be considered interchangeable in this discussion) in EABFT on the auction price. For example, the auctioned quantity of fresh BFT supplied by non-Japanese fleet doubled from 792 mt in 2012 to 1,632 mt in 2016. Over the same period, the average annual auction price for fresh BFT supplied by non-Japanese fleet decreased by 24.35%, from 3,434 yen/kg in 2012 to 2,864 in 2014 and further to 2,598 yen/kg in 2016. A similar downward trend is observed for the auction price of frozen BFT from 3,826 yen/kg in 2012 to 3,400 yen/kg in 2016.

### Simulated impact of increases in EABFT TAC in 2020 on the auction price in Japan

Given the integrated market and high substitutability between sashimi grade tuna products, to project the potential impact of the increases in the TAC of EABFT in 2020, the estimated scale flexibility from the SIDS demand system is used to capture the complexity of substitution across various sources of BFT supply and products forms.

The range of the shocks was specified to simulate an increase in the quota of EABFT to 30,000 mt or 40,000 mt in 2020 as EABFT quota scenarios #1 and #2, respectively. Under EABFT quota scenario #1, the annual global BFT landings will be 57,917 mt in 2020, i.e., 31.94% more than the global landings in 2014. If the eastern Atlantic BFT quota increases to 40,000 mt in 2020 under EABFT quota scenario #2, annual global BFT landings will reach 67,917 mt in 2020, a 54.73% increase from the global landings in 2014.

The percentage of EABFT accounting for global supply increased from 33.89% in 2014 to 49.99% in 2017. Under the EABFT quota scenarios #1 and #2, the percentage of EABFT accounting for global supply will increase further to 55.68% and 62.21% in 2020, respectively.

Table 6 shows how different sectors/fisheries would be affected by the TAC increases of EABFT by using the estimated scale flexibility to accommodate the potential of the substitution effect. This is to quantify the potential negative impacts on price of the proposed TAC in the retrospective status in 2014, 2017, and 2020, respectively.

**Table 6:** Simulation of the price response analysis of the bluefin tuna auction market in Japan and on various fishing fleets/sectors based on the shock of TAC of EABFT on global landings of BFT in 2017 and expected changes in 2020 (comparing to the base year in 2014).

Cases	Flexibility	Landings / TAC (mt)			Impact on fishing fleets/sectors
		2014 (Landings)	2017 (TAC)	2020 (Expected TAC)	
Shock: Increasing TAC of EABFT		13,500	23,655 (71.36% increase)	30,000~40,000 (301.2% Increase)	EABFT accounts for 33.89%, 49.99% and 55.68~62.21% of the global BFT landings in 2014, 2017, and 2020, respectively.
Impacts on global landings of BFT (comparing to the base year of 2014)		43,895	51,322 (16.92% increase)	57,917~67,917 (31.94%~54.73% increase)	High quality global BFT supplied increases 16.92% from 2014 to 2017, and 31.94%~54.73% from 2014 to 2020, depending on EABFT quota scenario.
**Impacts on global price (comparing to the base year of 2014)**					
**Product 1:** BFT_Fresh - Japanese fleet	-1.152		Prices drop by 19.49%	Prices drop by 36.80%~63.04%	Japanese fleets account for 33%, 26% and 21%~25% of global BFT landings in 2014, 2017 and 2020, respectively. Will experience a loss in total revenue, as the price for their products drops proportionally more than supply increases.
**Product 2:** BFT_Fresh - Non-Japanese Fleet	-1.004		Prices drop by 16.99%	Prices drop by 32.07%~54.94%	Non-Japanese fleets will experience a loss in total revenue, as the price for their products drops slightly proportionally more than supply increases.
**Product 3:** BFT_Frozen	-0.911		Prices drop by 15.41%	Prices drop by 29.10%~49.85%	Price goes down proportionally less than supply goes up, which may be beneficial to the EU fishermen who catch more than in previous years as a result of the overall quota increase. Note that absent individual quotas, there is no guarantee that this will be the case for individual fishermen. BFT landed by fishermen in either the Pacific or Western Atlantic will be worse off, as their quota will not be allowed to raise while they will still experience the price shock.
**Product 1a:** Atlantic western bluefin landings (supplied by Japanese or non-Japanese Fleet)	-1.152 or -1.004	1,626	2,000	2,250	While price is declining less than supply is increasing (proportionally), in 2015 and 2016 the West Atlantic BFT fishermen received no additional quota but a reduced price as a result of the EABFT supply shock. Thus, as a sector, the EABFT increase reduced their revenue. Note if the catch is landed by a non-Japanese vessel, then the scale flexibility will be -1.004 (as for Product 2 above).
**Product 1b:** Pacific bluefin (catch estimate from ISC)	-1.152 or -1.004	13,337	13,035	13,035	Pacific bluefin catch declined between 2014 and 2017, yet prices also dropped due to the EABFT supply shock (among other things), so Pacific bluefin fishing revenue decreased. The additional EABFT quota increase will further reduce prices, with no additional quota accruing to Pacific fishermen.
**Products 4 and 5:** SBT Fresh and Frozen Impacts on price (comparing to the base year of 2013)	-0.899 and -0.900	14,647	14,637	14,637	While price is declining less than supply is increasing (proportionally) for the entire BFT market/substitutes, the SBT quota was stable at 14,637 between 2015 and 2020, and price was declining at that time as a result of the EABFT supply shock. Thus, SBT was worse off as a sector—fresh and frozen—during this time.
			Prices drop by 15.21%	Prices drop by 28.72%~49.20%	
**Product 6:** Bigeye Fresh	-1.224		Prices drop by 20.71%	Prices drop by 39.10%~66.98%	Will experience a loss in total revenue, as the price for bigeye products globally decreases if the total combined supply of bigeye and BFT products increases.
Atlantic bigeye landings/TAC		67,986	65,000	65,000	

### Do higher landings guarantee higher revenue for EABFT fisheries?

#### Hypothesis testing (1): Impacts on the price of fresh BFT landed by Japanese Fleets

The scale flexibility of fresh BFT supplied by Japanese fleets from either the Atlantic or Pacific Ocean is estimated to be over 1, at the 5% significance level for a one-sided hypothesis test. This means total revenue will in fact decrease slightly, with a price that drops proportionally more than the supply increases. Given that any increase in global BFT landings will consist mainly of landings associated with the increased TAC for EABFT, the auction price for fresh BFT landed by the Japanese fleet is projected to decline by 36.80% and 63.04% in 2020 under EABFT quota scenarios #1 and #2, respectively. If some of these Japanese fleets are constrained by fishing area and cannot increase their landings of fresh BFT from the Eastern Atlantic (for example, for vessels that operate exclusively in the Pacific), they will suffer negative price effects and see their revenue fall as a direct result of the EABFT quota shock.

#### Hypothesis testing (2) Impacts on the price of fresh BFT landed by Non-Japanese Fleets

The scale flexibility of fresh BFT supplied by non-Japanese fleets is not significantly different from 1, which means total the revenue likely remains the same with an increase in global supply of BFT. Once again, however, if those fishermen are constrained by any inability to increase their landings of fresh BFT (for example, if they do not have the ability to fish in the Eastern Atlantic), they will also incur negative price effects.

#### Hypothesis testing (3) Impacts on the price of frozen BFT

Only frozen BFT exhibits a scale flexibility of less than unity (-0.911) under a 5% significance level for one-sided hypothesis test, though it is close to 1 (unity). Thus, given a 1% increase in global supply of BFT, the price will drop by 0.911%, and total revenue will increase overall by just 0.089%, implying a slight gain in total revenue if they are able to increase their landings of EABFT. Note (again) that this result only applies to those landing bluefin from the Eastern Atlantic; those unable to acquire any increased landings will simply see a decline in price.

While the frozen EABFT sector may be the only “winner” if potential EABFT supply increases come to pass (given, again, that price still decreases, just at less of a rate than supply increases), there is absolutely no guarantee that an individual fishermen’s revenue will go up, as the absence of individual harvesting rights prevents any individual from a guaranteed share of the TAC/catch. Additionally, the risk to the health of the stock and the number of “losers” may outweigh any net benefit accrued by the frozen EABFT sector. Fishermen of Japanese BFT, western bluefin, Atlantic and Pacific bigeye and SBT, and exporters of fresh BFT all likely stand to lose. Furthermore, supply chain characteristics texture the small benefit enjoyed by the fresh EABFT sector.

The 2016 EU trade data (EUMOFA) shows that EABFT exports from the EU totaled 15,760 mt and that 78.3% of these exports were sold fresh and, most importantly, 80.0% of the EU’s EABFT exports to Japan were fresh (a loser under the demand system specified). Thus, there is a strong argument that a solid portion of the EU industry/export sales, overall, is harmed financially by the increased supply of EABFT in recent years.

In summary, the price for fresh BFT is more responsive than the price for frozen BFT. There is a concern from the industry about the impact of the increase in landings—potentially by 31.94% to 54.73% in 2020 under EABFT quota scenarios #1 and #2, respectively—compared to the base year of 2014. Such increases will drive the BFT export value from the EU to reflect the likely impact to different sectors outlined above. Since 80% of the BFT exports to Japan are fresh and only 20% are frozen, only those exporting the relatively smaller proportion of frozen BFT may see any positive change in export value. However, the losses to the 80% of harvesters who export fresh BFT will certainly not be compensated by the gains to the 20% of those who export frozen BFT, raising important questions about the objectives of state trade policies.

Because ranching activities mean the surface purse-seine fleet’s impact can add an additional 30–60% increase in the quantity of tuna at the auction market than countries with other types of fishing gear, the impact they have on global prices may be somewhat magnified, depending on how their quota is utilized. If the surface purse seine fleet uses the EABFT quotas for ranching, the purse seine fleet will add more pounds at the auction per % increase in quota than other sectors. That is also the case for SBT quota used by Australia surface purse-seine fleet to cage culture juvenile SBT, so there is a need to calculate whole round equivalent quota weight auctioned in Japan.

#### Hypothesis testing (4) Impacts on the price of fresh and frozen southern bluefin tuna

The fresh SBT exhibits a scale flexibility that is no different than unity (-0.899) at a 5% significance level, but the frozen SBT exhibits a scale flexibility of less than unity (-0.900) under a 5% significance level for one-sided hypothesis test, albeit close to one. Currently, the SBT TAC is set to remain at 14,637 mt between 2015 and 2020, and thus the price is projected in this study to decline between 28.72% and 49.20%, if the EABFT supply shock continues through 2020. Thus, SBT will be worse off, whether fresh and frozen, due both to the price elasticities as well as declining prices caused by the increases in quota from another region.

#### Hypothesis testing (5) Impacts on the price of fresh bigeye tuna

Fresh bigeye tuna auctioned in Tokyo Market might be supplied by landings from the Pacific, Indian and Atlantic Oceans. Either the bigeye tuna’s global landings or landings in each of the oceans have all trended down since 2000. The aggregated landings, which peaked at 533,192 mt in 2000, declined to 376,360 in 2010 [50]. The Atlantic bigeye industry has already been subject to significant losses due to the decrease in landings from 85,865 mt in 2011 to 67,986 mt in 2014, with an estimated 67% chance that the stock had been overfished and with overfishing still occurring in 2014 [51]. Even if the quota is stabilized as expected at the lower level of 65,000 mt in 2020, the auction price of fresh bigeye is projected to decline by 39.10% to 66.98% in 2020 due to the EABFT quota shock ranging from 30,000 mt to 40,000 mt under scenarios #1 and #2, respectively. In this case, whether the bigeye tunas are supplied from the Pacific, Indian or Atlantic Oceans, their revenue will all decline dramatically.

## Discussion and concluding remarks

This study presents the first comprehensive view of the global BFT market and the incentives created for conservation and management of regional and global BFT by changes in supply and through the management of quotas. We simulate the response of BFT prices and revenues through changes in supply by using the price and scale flexibilities estimated from an inverse demand system based on data from the central auction seafood auction market in Tokyo Market. Because the quantities, and not the prices, of fish closely related in demand are held constant, the price flexibilities (quantity elasticities) account for adjustments in related markets, i.e. the flexibilities have a general equilibrium interpretation.

The results show several broad patterns. First, when there are increases in aggregate bluefin (and bigeye) tuna on the global market, the prices of two major bluefin products decline proportionately more and lead to a loss in revenue, two product prices decline proportionately less and lead to a rise in revenue, and two product prices decline proportionately the same and lead to no change in revenue (scale flexibilities larger, less than, or equal to one, respectively). Second, product prices are inflexible to changes in their own supply, i.e. these prices demonstrate responses proportionately smaller than the quantity changes (in other words, their own-price flexibilities of inverse demand are inflexible). This inflexibility implies that the corresponding price elasticities of direct demand are elastic (i.e. that quantity demanded is highly responsive to own price changes) due to the generally high substitution of products in direct demand. Third, the price for fresh product is more responsive than the price for frozen product (seen in both BFT and bigeye) in the Tokyo Market when aggregate supply changes (i.e. frozen scale flexibilities smaller in absolute value than fresh products).

Fourth, buyers further distinguish fresh from frozen products, as indicated by the estimated scale flexibilities for both frozen BFT (-0.91) and frozen southern BFT (-0.90) are significant less than unity, and more inflexible than those of fresh bluefin tuna products. Fifth, buyers in the Tokyo Market distinguish Japanese producers’ BFT from the non-Japanese producers’ BFT on several fronts: (1) the scale flexibility for fresh BFT from Japanese producers is the only flexible BFT scale flexibility (bigeye’s is also flexible, but it is not BFT); (2) the own price of fresh BFT from Japanese producers, while not very responsive to changes in its own quantity, is still more responsive than the own price responsiveness of other BFT producers to changes in their own quantities (the own-quantity price flexibility for fresh BFT from Japanese producers, while inelastic, is larger than the inelastic own-quantity price flexibilities for other BFT products); (3) the other products are not considered to be close p-substitutes in direct demand (as indicated by the large positive reciprocals of the price flexibilities); and (4), the relative price of Japanese producer supplied BFT to other BFT prices is more responsive to changes in the quantities supplied of other products (as indicated by larger positive Morishima Elasticities of Complementarity than for other product forms).

Sixth, relative prices for two different products, even for Japanese producer-supplied BFT, are comparatively stable with changes in supply of one of the other products (as indicated by small and positive Morishima Elasticities of Complementarity). Seventh, the market for all three species of BFT is integrated and is considered highly substitutable between different forms (fresh/frozen and Japanese/non-Japanese) and species of BFT (small and positive Morishima Elasticities of Complementarity). Prices and quantities of other species and product forms absorb any small shocks due to sudden changes in a species price. This absorption smooths out market responses and stabilizes consumer welfare. And finally, eighth, regional context matters in a globally integrated market; as shown in this study, even a scale flexibility less than unity can result in lost revenue, if the quota increases are captured entirely by a different region or sector.

Unfortunately, these results imply that there is little incentive for individual regional suppliers of bluefin tuna, or even individual tuna-Regional Fisheries Management Organizations (t-RFMOs) that manage the fisheries, to individually reduce their catches for either wild caught or ranched bluefin tuna, as this would lower their revenue due to the inflexibility of their own-quantity price flexibilities. In fact, the individual regional incentives across the board are to increase supply and Total Allowable Catches to increase revenue. However, this is not the case if the total supply of all product forms and species increase in four of the six cases considered here: all fresh products, because the unitary or flexible scale flexibilities, imply price responses proportional–or even more than proportional–to reductions in total supply, at most maintaining and at worst increasing fishermen’s revenue and operating profits. Put another way, the global incentives for these four cases of fresh product forms (two of which are staples of the Tokyo Market) are to reduce supply to increase operating profit. In the other two cases, both of which are frozen product forms and luxury goods in the Tokyo Market, increases in aggregate supply will raise revenue and reductions in aggregate supply will lower revenue.

In sum, uniform reduction in aggregate supply through TAC reductions creates conflicting incentives for different actors the short run (setting aside, for the moment, differing fleet abilities to take advantage of increased quotas). Short-run incentives for individual BFT stocks counter conservation for some stocks. Reductions in aggregate supply and conservation for the four fresh products create positive short-run conservation incentives through no loss in revenue and increases in operating profit, but concomitantly create negative short-run conservation incentives for the stocks that support the two frozen products. Eventually, reductions in catch and supply would allow stocks to rebuild and total revenues to climb for all BFT species, but short-run incentives through revenues do not uniformly support this approach through RFMOs.

The price response of the global BFT market to an exogenous supply shock from one region justifies the need to call for consistent management measures across all the Regional Fisheries Management Organizations together [52]. When talking about a globally-traded commodity such as highly migratory tuna, localized decisions (such as those in just one ocean) that would restrict allowable catch does not lead to the same responses and create the same incentives for stakeholders in other regions–despite many of these stakeholders in different regions ostensibly representing the same overall economic interest of the same countries, just in different fora. The mixed requirements of increased, constant, or decreased aggregate supply for different products from different fisheries managed by different RFMOs, and the consequent requirement for coordination across RFMOs to create positive conservation incentives, makes this a difficult task indeed.

That economic incentives count when conserving renewable resources and managing fisheries is beyond doubt. However, the primary focus so far has been on incentives created by property rights. The relationship between TACs (or any other total production cap) and revenues and profit, all conjoined by globally linked prices, has not received sufficient attention. First, local fishermen’s revenue and license fees collected by coastal states can fluctuate substantially because of higher catches in other tuna fisheries elsewhere in the world. The developing economies that are highly dependent on tuna fisheries could be strongly affected [53][7]. Second, the investment cycle, and the resulting dynamics of the fleet, might be influenced with lagged effects both by the availability of resources and price levels. If fishermen could collectively lower their operating cost through a quota-trading mechanism monitored by the tuna RFMOs, for example, they would be in a better position to contribute to the conservation effort by avoiding overcapacity and “the race to fish.” Setting aside the context of RFMO negotiations, the tuna fishing industry would still benefit by recognizing that a stable global supply for bluefin tuna helps the industry maintain total landing values in the long run, since increases in quantity could be more than offset by decreases in price.

Is there another way to create conservation incentives as an alternative to globally coordinated reductions in TACs by all of the t-RFMOs? One option is to alter the focus from producers’ revenues to producers’ profits, through reducing capacity and thereby reducing operating and fixed costs and leaving more bluefin tuna available to each producer. Higher producer profits from reduced capacity and lower fixed and operating costs would compensate for lower overall revenues. Other than bankruptcy or voluntary exit from the fisheries, some form of individual or group right, such as a transferable quotas or limits on catch or effort, provides one of the few other methods by which to reduce capacity and hence increase profits. Many tuna fisheries are comprised of multi-vessel companies, and transferable quotas or limits would allow these companies to reallocate fishing opportunities among their vessels and even remove vessels from production.

Rights could also lower the comparatively high private discount rate, created by the “Tragedy of the Commons” and the race to fish under the absence of property rights, to a lower social discount rate, thereby boosting the net present value of higher future revenues and catches that eventually follow through rebuilding stocks with higher biomass and numbers. Moreover, the long-lived and slow growing life history of BFT aggravates the incentives from the current high private discount rate to emphasize current catches at the expense of larger future catches, which require the rebuilding of stocks through lower current production. The other main alternative to reduce capacity, and thereby increase producer profits (to counter the lower immediate revenues from conservation), is vessel buybacks; however, these are ineffective without first altering the incentives away from the “race-to-fish” in the absence of property rights [54]. A different type of approach allocates TAC across Contracting Parties to the Conventions, which several tuna and non-tuna RFMOs follow [55]. However, allocating TAC across RFMO CPCs merely shifts the “race-to-fish” incentives down to the CPC level from the RFMO level, and unless individual CPCs introduce incentive-based policies, nothing substantive changes [56]. The Commission for the Conservation of Southern Bluefin Tuna (CCSBT) is the apparent exception, due to the small number of CPCs and the composition of the CPCs, both of which collectively favor property rights, profits, and conservation.

Society draws many benefits from the existence of healthy tuna stocks, including non-market economic values for biodiversity, existence, and ecosystem services. Unless and until conservation-oriented actions begin to pay short-term benefits for fishermen who receive direct use value from tuna in the form of profits, however, the current incentives fishermen face seem unlikely to motivate support for a reduction in bluefin tuna quota. This study, however, raises the notion that the price response within the global bluefin sashimi market itself could potentially provide the short-term incentives needed to achieve dual management goals of a sustainable industry and a healthy tuna population. With the multilateral cooperation among nations required for self-enforcing through the international tuna bodies likely remaining elusive, an alliance of economic and conservation-motivated stakeholders may just be bluefin tuna’s best shot at recovery.

## Supporting information

S1 Appendix Model specification of general synthetic inverse demand system (GSIDS). (DOCX)

S2 Appendix Table A. Monthly sample statistics of tuna auction market in Tokyo, Japan.
(XLSX)
