# Supplementary material for: More landings for higher profit? Inverse demand analysis of the bluefin tuna auction price in Japan and economic incentives in global bluefin tuna fisheries management
Source: PLoS One. 2019 Aug 23;14(8):e0221147. doi: 10.1371/journal.pone.0221147 (PMC6707606; doi:10.1371/journal.pone.0221147)
Supplement: S1 Appendix — (DOCX) [file pone.0221147.s001.docx]

**S1 Appendix. Model Specification of General Synthetic Inverse Demand System (GSIDS)**

Follow by equation (4), the GSIDS can be written as:

*w_it_* d ln *π_it_ =* (*h_i_ - d_1_ w_it_*) dln *Q +* Σ_j_ (*h_ij_ - d_2_ w_it_ (δ_ij_ -w_jt_*)) d ln *q_jt_* (a1)

where, for time *t*, *π_it_ = p_it_/m_t_* is the normalized price of good *i* such that with *p_it_* is price and *m_t_* is total expenditure; *q_it_* is quantity of good *i; w_it_* = *q_it_π_it_* is budget share of *q_it_*; d ln *π_it_ =* log(*π_it_/π_it-1_*); d ln *q_it_ =* log(*q_it_/q_it-1_*); *δ_ij_* is Kronecker delta such that *δ_ij_* = 1 if *i* = *j* and *δ_ij_* = 0 otherwise; and dln Q = Σ*_j_w_jt_ d ln q_jt_* is the Divisia volume index.

Suppress subscript *t* for parsimony in what follows. The above inverse-demand system satisfies $\sum_{i} h_{i}=-1+d_{i}$ and $\sum_{i} h_{ij}=0$ (adding-up), $\sum_{j} h_{ij}=0$ (homogeneity), and $h_{ij}=h_{ji}$ (Antonelli symmetry). Note that the adding-up restriction $\sum_{i} h_{i}=\sum_{i} w_{i}f_{i}=-1$ is based on the reference quantity vector or the reference quantity vector which has a scale factor *k* = 1 (Anderson and Blundell 1983). The scale and compensated quantity flexibilities for (a1) are,

1. **Scale Flexibility**

The scale flexibility is calculated as:

*f_i_ =h_i_ / w_i_ - d_1 ._* (a2)

1. **Own-quantity and Cross-quantity Flexibility**

The compensated cross-quantity flexibility is calculated as:

*f_ij_* = h_ij_/ w_i_ - d_2_(δ_ij_ - w_j_)*  *_._* (a3)

Nested specification and their flexibilities can be obtained by restricting *d_l_* and *d_2_* in (a1), (a2) and (a3); that is,

*d_l_* = 0, *d_2_* = 0 for the Rotterdam Inverse Demand System (RIDS);

*d_l_* = 1, *d_2_* = 0 for the Laitinen-Theil model, known as Inverse Census Bureau of Statistics (ICBS) Model;

*d_l_* = 1, *d_2_* = 1 for the Almost Ideal Inverse Demand System (AIIDS);

*d_l_* = 0*, d_2_* = 1 for the RAIIDS model with RIDS scale effects and AIIDS quantity effects, known as Inverse National Bureau of Research (INBR).

These parametric restrictions can be tested for model comparison because all of the parameters in each of various model specifications are estimated with a maximum likelihood method maximization, models can thus be compared according to quality of estimations. Related parameters and those indicators used in the results part, such as the Antonelli matrix, or the Morishima elasticities.

The Antonelli matrix *H* = [*h_ij_*] reflects the degree of interactions among goods in their ability to satisfy demand. More of good *i* is generally sold at a lower price for *i*. One may also say that a good is its own substitute. Extending the notion of substitution to all negative *h_ij_*, it is natural to consider a positive *h_ij_* as an indication of complementarity between *i* and *j*. Note that the adding-up condition Σ_i_ *h_ij_* = 0 together with *h_jj_* < 0 means that Σ_i≠j_ *h_ij_* > 0; therefore, for i ≠ j complementarity may dominate in an inverse-demand system, that is, each row of the Antonelli matrix must sum to zero because of the property of homogeneity in the system of budget share equations and a good, being a “substitute for itself,” has a negative own-price flexibility. As a result, complementarity dominates over substitutability in the off-diagonal terms, i.e., the cross effects in the Antonelli matrix are biased toward complementarity. This dominance does not come from the structure of preferences but from the condition π’x = 1, which makes the *h_ij_*s imperfect measures of the interaction of goods in their satisfaction of wants (Chiang *et al*. 2001). Barten and Bettendorf (1989) worked with a transformation of the *h*-vector. Using the vectors *h =* [*h_i_*] and *w =* [*w_i_*], they derived the counterpart of the Allais coefficients for the inverse demand system. By selecting *r* and *s* as the standard pair of goods, the Allais coefficient for the inverse demand system can be defined as:

*a_ij_* = *h_ij_/w_i_w_j_ - h_rs_/w_r_w_s_ +* (*h_i_/w_i_ – h_r_/w_r_*) *+* (*h_j_/w_j_ – h_s_/w_s_*) (a4)

In the definition of *a =* [*a_ij_*], the subscripts *r* and *s* refer to a standard pair of goods *r* and *s*. The above equation indicates that *a_rs_ = 0*. Thus *a_ij_ > 0* indicates that *i* and *j* have the same type of interaction as *r* and *s*. Based on the Allais coefficient, the measure of the intensity of interaction can be defined as:

*α_ij_ = a_ij_/*(*a_ii_a_jj_*)*^1/2^*  (a5)

which for a negative definite matrix *A =* [*a_ij_*], *a_ij_* varies between –1 (perfect substitution) and +1 (perfect complementarity).

All of the data used in the model are compiled from the monthly average tuna auction price and cumulative monthly quantity from January 2003 to December 2016, with more than three transactions across more than three dealers and sellers at Tokyo Metropolitan Central Wholesale Market (Abbreviated as “Tokyo Market” in the paper), and cannot be de-identified for any individual information.

1. **Morishima Elasticity of Complementarity (MEC)**

The Morishima elasticity of complementarity could serve as a standardized measure of substitutability as an adequate measure of interaction between commodities. The inverse demand system analogue of the Morishima elasticity of substitution (Blackorby and Russel 1989) is more useful than the elasticities themselves (Park, Thurman and Easley 2004). The MEC is defined as s_ij_ = f_ji_* - f_ii_* to represent the proportionate change in the *j,i* compensated demand price ratio due to a 1% increase in the *i*th quantity. The elasticity shows how the (shadow) price ratio changes and takes positive value values for q-complements and negative values for q-substitutes.

The Morishima elasticities of complementarity, reported in S1 Table, represent the proportionate change in the *j*th over *i*th compensated demand price ratio due to a 1% increase in the *i*th quantity. The elasticities of complementarity are all positive, reflecting complementarity and implying negative own-quantity elasticities. All values are inelastic. The inelastic and positive estimate of 0.288 implies, for example, a 1% increase in the quantity of frozen BFT results in a 0.288% increase in the ratio of frozen BFT price to the fresh Japanese fleet’s BFT price, all other quantities held constant.

|  | BFT_Fresh Japanese Fleet | | BFT_fresh Non-Japanese fleet | | BFT Frozen | | SBT Fresh | | | SBT Frozen | | Bigeye Fresh | |
| --- | --- | --- | --- | --- | --- | --- | --- | --- | --- | --- | --- | --- | --- |
| BFT_Fresh Japanese fleet | 0.000 |  | 0.133 |  | 0.114 |  | 0.136 |  | 0.320 | |  | | 0.000 |
| BFT_Fresh Non-Japanese fleet | 0.223 |  | 0.000 |  | 0.084 |  | 0.148 |  | 0.216 | |  | | 0.353 |
| BFT_Frozen | 0.288 |  | 0.184 |  | 0.000 |  | 0.151 |  | 0.188 | |  | | 0.353 |
| SBT_Fresh | 0.225 |  | 0.128 |  | 0.073 |  | 0.000 |  | 0.208 | |  | | 0.371 |
| SBT_Frozen | 0.338 |  | 0.161 |  | 0.059 |  | 0.136 |  | 0.000 | |  | | 0.520 |
| Bigeye Fresh | 0.247 |  | 0.157 |  | 0.082 |  | 0.061 |  | 0.284 | |  | | 0.000 |

**S1 Table A. The Morishima Elasticities of Complementarity**

Note: MEC represents the proportionate change in the *j*th over *i*th compensated demand price ratio due to a 1% increase in the *i*th quantity. MEC > 0 signifies products are q-complements. 0 < MEC < 1 signifies inelastic complementarity.
